# Supplementary figures and images for: Interlaboratory comparison for the Filovirus Animal Nonclinical Group (FANG) anti-Ebola virus glycoprotein immunoglobulin G enzyme-linked immunosorbent assay
Source: PLoS One. 2020 Aug 25;15(8):e0238196. doi: 10.1371/journal.pone.0238196 (PMC7447032; doi:10.1371/journal.pone.0238196)

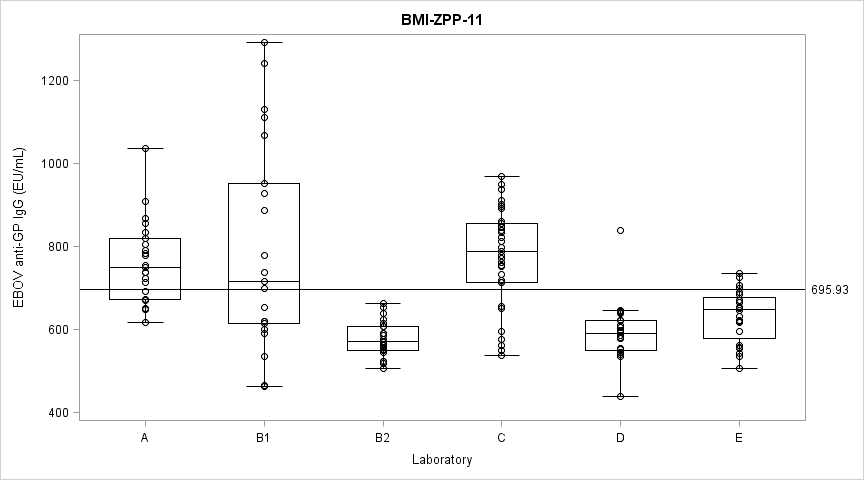

Supplement: S1 Fig — (Consensus Concentration = 695.93). Center line in the box depicts the median concentration while the height of the box represents the 25th and 75th percentile of the concentration distribution. Vertical lines extending above and below the box represent the maximum and minimum concentration values for the laboratory. Open circles show the observed concentrations. (TIF) [file pone.0238196.s001.tif]

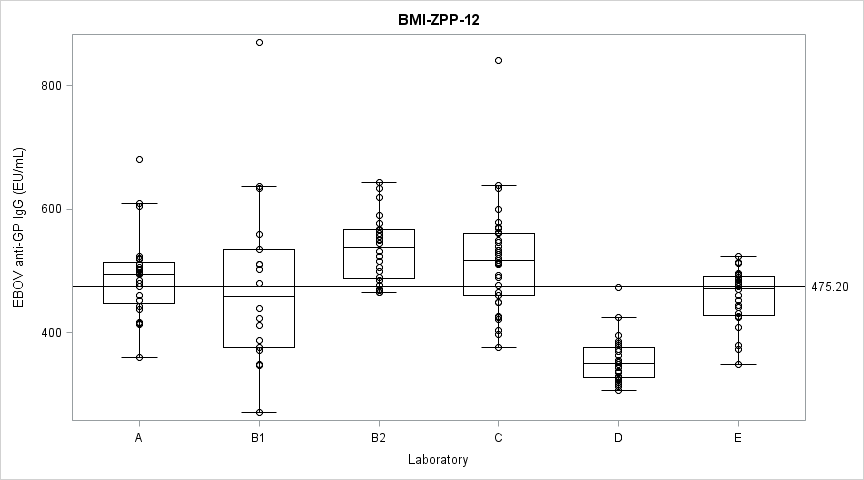

Supplement: S2 Fig — (Consensus Concentration = 475.20). Center line in the box depicts the median concentration while the height of the box represents the 25th and 75th percentile of the concentration distribution. Vertical lines extending above and below the box represent the maximum and minimum concentration values for the laboratory. Open circles show the observed concentrations. (TIF) [file pone.0238196.s002.tif]

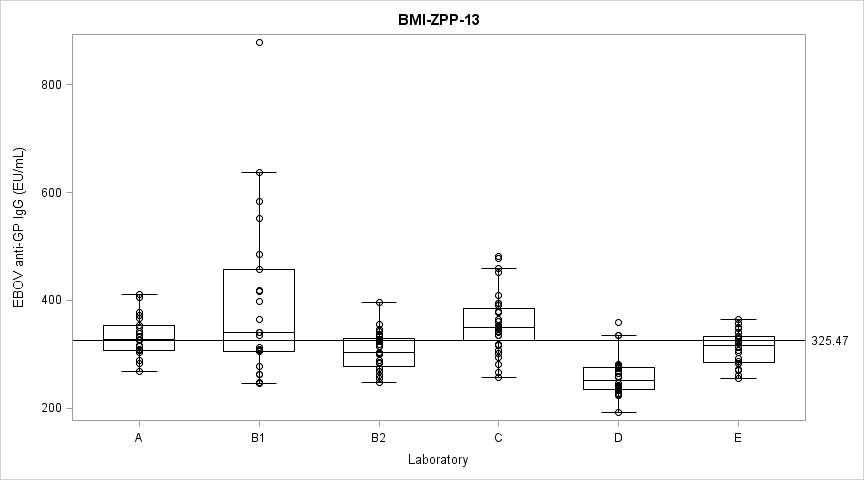

Supplement: S3 Fig — (Consensus Concentration = 325.47). Center line in the box depicts the median concentration while the height of the box represents the 25th and 75th percentile of the concentration distribution. Vertical lines extending above and below the box represent the maximum and minimum concentration values for the laboratory. Open circles show the observed concentrations. (TIF) [file pone.0238196.s003.tif]

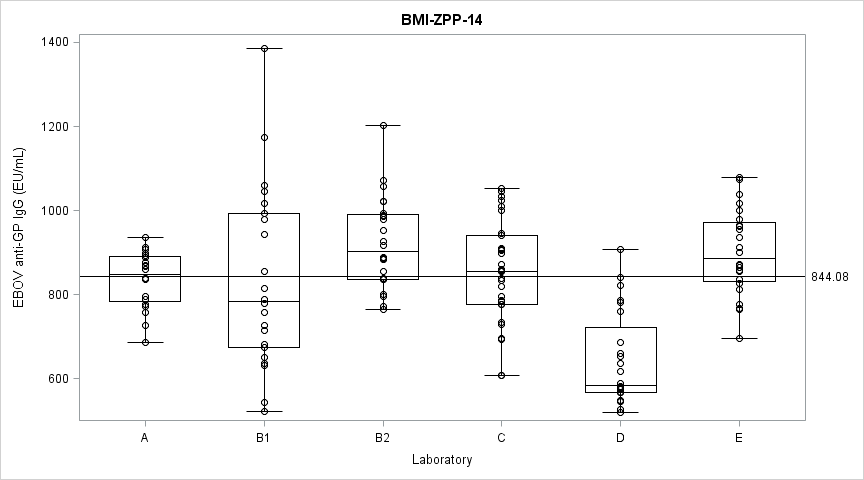

Supplement: S4 Fig — (Consensus Concentration = 844.08). Center line in the box depicts the median concentration while the height of the box represents the 25th and 75th percentile of the concentration distribution. Vertical lines extending above and below the box represent the maximum and minimum concentration values for the laboratory. Open circles show the observed concentrations. (TIF) [file pone.0238196.s004.tif]

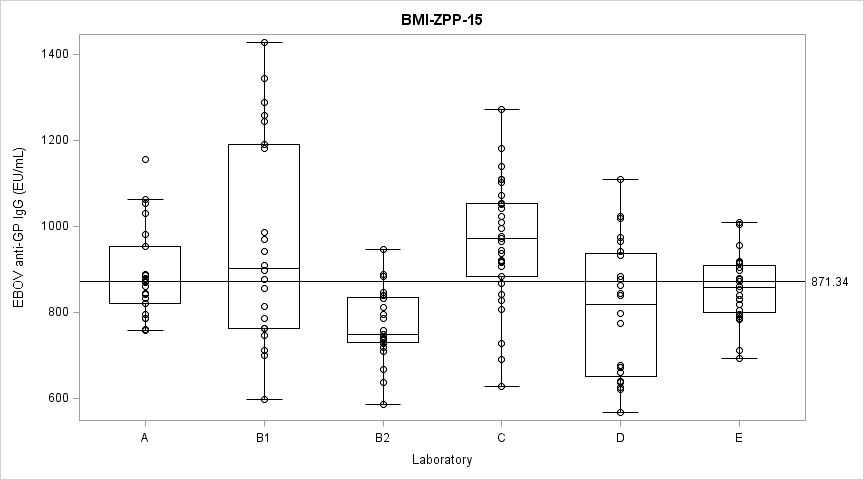

Supplement: S5 Fig — (Consensus Concentration = 871.34). Center line in the box depicts the median concentration while the height of the box represents the 25th and 75th percentile of the concentration distribution. Vertical lines extending above and below the box represent the maximum and minimum concentration values for the laboratory. Open circles show the observed concentrations. (TIF) [file pone.0238196.s005.tif]

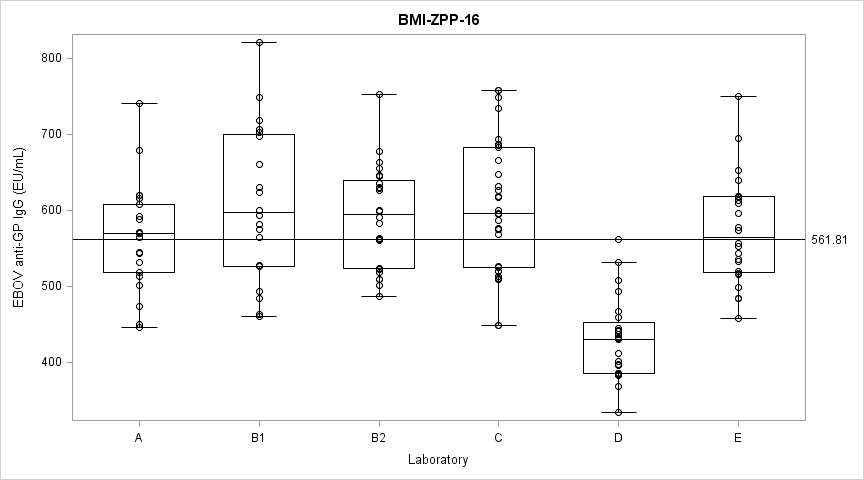

Supplement: S6 Fig — (Consensus Concentration = 561.81). Center line in the box depicts the median concentration while the height of the box represents the 25th and 75th percentile of the concentration distribution. Vertical lines extending above and below the box represent the maximum and minimum concentration values for the laboratory. Open circles show the observed concentrations. (TIF) [file pone.0238196.s006.tif]

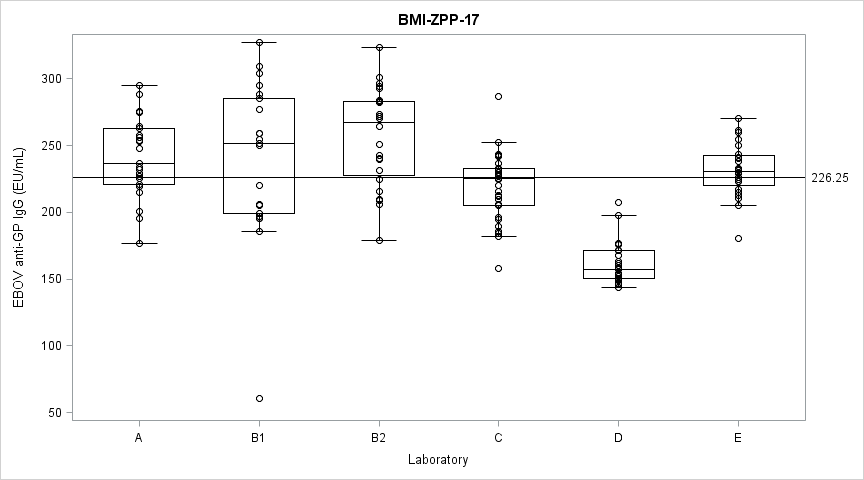

Supplement: S7 Fig — (Consensus Concentration = 226.25). Center line in the box depicts the median concentration while the height of the box represents the 25th and 75th percentile of the concentration distribution. Vertical lines extending above and below the box represent the maximum and minimum concentration values for the laboratory. Open circles show the observed concentrations. (TIF) [file pone.0238196.s007.tif]

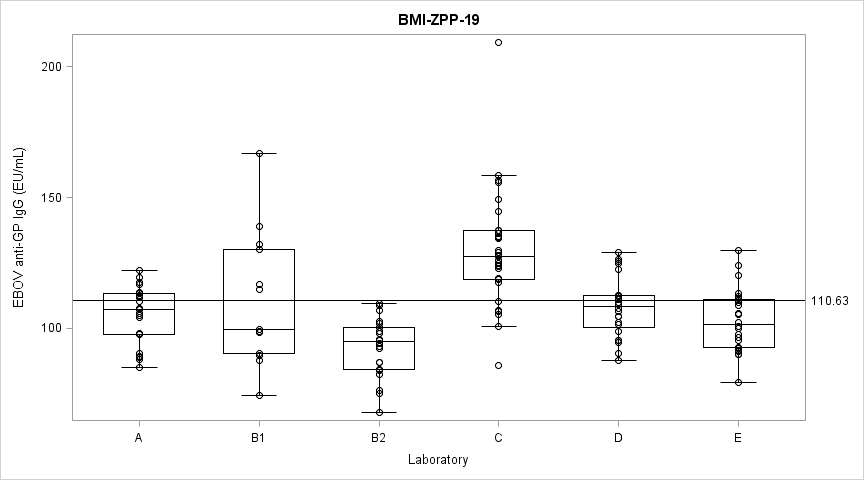

Supplement: S8 Fig — (Consensus Concentration = 110.63). Center line in the box depicts the median concentration while the height of the box represents the 25th and 75th percentile of the concentration distribution. Vertical lines extending above and below the box represent the maximum and minimum concentration values for the laboratory. Open circles show the observed concentrations. (TIF) [file pone.0238196.s008.tif]

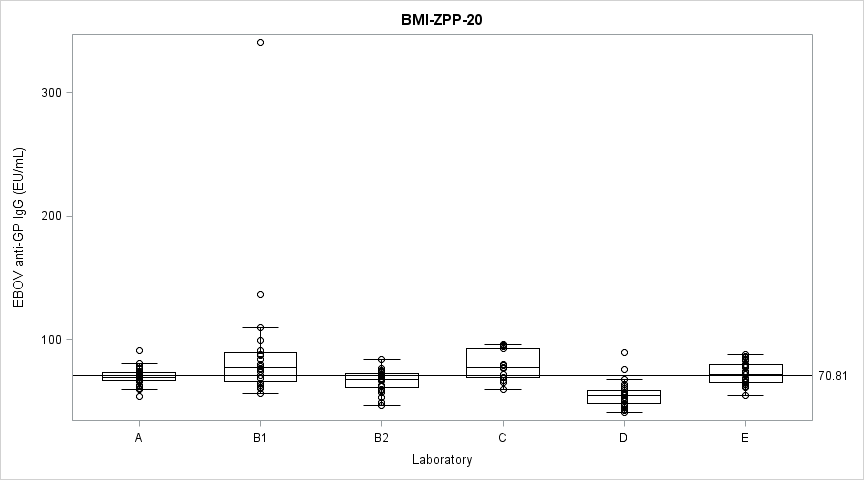

Supplement: S9 Fig — (Consensus Concentration = 70.81). Center line in the box depicts the median concentration while the height of the box represents the 25th and 75th percentile of the concentration distribution. Vertical lines extending above and below the box represent the maximum and minimum concentration values for the laboratory. Open circles show the observed concentration. (TIF) [file pone.0238196.s009.tif]
